# The role of beta-2-glycoprotein I in health and disease associating structure with function: More than just APS

**DOI:** 10.1016/j.blre.2019.100610

**Published:** 2020-01

**Authors:** Thomas McDonnell, Chris Wincup, Ina Buchholz, Charis Pericleous, Ian Giles, Vera Ripoll, Hannah Cohen, Mihaela Delcea, Anisur Rahman

**Affiliations:** a Rheumatology, Division of Medicine, Rayne Institute, University College London, UK; b Nanostructure Group, Institute of Biochemistry, University of Greifswald, Germany; c Imperial College London, Imperial College Vascular Sciences, National Heart & Lung Institute, ICTEM, Hammersmith Campus, Du Cane Road, London, UK; d Department of Haematology, University College London Hospitals NHS Foundation Trust, London, UK

**Keywords:** Beta-2-glycoprotein I, Antiphospholipid syndrome, Coagulation, Complement, Structural biochemistry

## Abstract

Beta-2-Glycoprotein I (β2GPI) plays a number of essential roles throughout the body. β2GPI, C-reactive protein and thrombomodulin are the only three proteins that possess the dual capability to up and down regulate the complement and coagulation systems depending upon external stimulus. Clinically, β2GPI is the primary antigen in the autoimmune condition antiphospholipid syndrome (APS), which is typically characterised by pregnancy morbidity and vascular thrombosis. This protein is also capable of adopting at least two distinct structural forms, but it has been argued that several other intermediate forms may exist. Thus, β2GPI is a unique protein with a key role in haemostasis, homeostasis and immunity. In this review, we examine the genetics, structure and function of β2GPI in the body and how these factors may influence its contribution to disease pathogenesis. We also consider the clinical implications of β2GPI in the diagnosis of APS and as a potentially novel therapeutic target.

## 1 Introduction

### 1.1 What is beta-2-glycoprotein I (β2GPI)?

Beta-2-Glycoprotein I (β2GPI) is a unique five domain protein comprising four similar complement control protein (CCP)-like domains (DI-DIV) and one different domain (DV) with a large lysine loop (1C1Z, Ensemble). It is a soluble blood protein with a circulating concentration of 0.2 mg/ml [[1], [2], [3]] and a molecular weight of 48 kDa. β2GPI has many proposed functions and roles within the body including the regulation of complement and haemostasis. Furthermore, it contains the main antigenic target of pathogenic autoantibodies found in patients with the autoimmune disorder antiphospholipid syndrome (APS). Often β2GPI is only considered important in the context of APS; however, in this review we will be examining its wider functions in physiology and pathology.

### 1.2 What is antiphospholipid syndrome (APS) and how is β2GPI important?

APS is an autoimmune disorder in which autoantibodies cause thrombosis and/or recurrent miscarriage or other obstetric morbidity. Although these antibodies are generally termed antiphospholipid antibodies (aPL), this term is a misnomer because the pathogenic antibodies in APS target proteins that associate with PL, the most important of which is β2GPI.

Antibodies of the IgG or IgM isotype to β2GPI are one of the three criteria antibodies in APS diagnosis [4]; the others are the lupus anticoagulant (LA) assay and the anti-cardiolipin (aCL) assay. aCL from patients with APS (but not from non-APS patients) require β2GPI as a co-factor for CL-binding whilst the LA effect has been shown to be β2GPI-sensitive in these patients [[5], [6], [7]]. Thus, both the aCL and LA assays may indirectly be dependent on the function and structure of β2GPI.

APS is estimated to affect between 0.3-1% of the population[8]. However, a recently published population based study assessing the epidemiology of APS suggested the figure may be lower, around 50 per 100,000 people [9]. Overall, APS carries significant morbidity and is a leading cause of strokes in people under 50 years old [8]. Andreoli *et al* estimated that APS may be a contributory factor in 6.1% of cases of pregnancy morbidity, 13.5% of strokes, 11.5% of myocardial infarctions and 9.5% of deep vein thromboses [10].

Some debate exists within the field regarding the potential to subdivide patients into thrombotic or obstetric subgroups. Traditionally this has been difficult to achieve, particularly because many patients suffer both thromboses and pregnancy loss. However, in recent years research has begun to separate the properties of antibodies found in these two groups of patients. Ripoll and Poulton have both shown differential cellular effects by antibodies from obstetric and thrombotic patients [11,12]. Ripoll et al showed distinct molecular signatures were detected by gene array when comparing monocytes exposed to IgG from patients suffering thrombotic or obstetric APS [12]. In a similar vein, Poulton et al showed that purified IgG from patients with obstetric but not thrombotic manifestations of APS were capable of inhibiting trophoblast invasion in an *in-vitro* assay [11]. Groups have also suggested different pathophysiological mechanisms drive the two variants of disease with causes of obstetric pathogenesis including deficient endometrial angiogenesis, inhibited toll-like receptors on trophoblasts and altered trophoblast interleukin-8 secretion [[13], [14], [15], [16], [17]]. Despite this research, the idea of two distinct syndromes is still somewhat controversial in the field. A comprehensive review was recently published by Meroni *et al* in 2018 [18].

Current therapies for APS are very limited. The only evidence-based treatment known to reduce the risk of recurrent thrombosis is long-term anticoagulation [19]. This form of therapy has most commonly been achieved using warfarin or other vitamin K antagonists (VKAs), although direct oral anticoagulants such as rivaroxaban are now coming into use. A non-inferiority trial in the United Kingdom, that used a laboratory surrogate primary outcome, concluded that rivaroxaban offers a potentially effective, safe and convenient alternative to warfarin in APS patients with venous thromboembolism requiring standard intensity anticoagulation [20] though it should be noted that there were no thrombosis in either arm of the study. In contrast, a more recent Italian study was discontinued due to excess adverse events (including myocardial infarction, stroke and bleeding) in the rivaroxaban arm, versus standard intensity warfarin [21]. This study was limited to triple aPL-positive (anti- β2GPI, aCL and LA positive) thrombotic APS patients, a high-risk group in which the same authors previously reported recurrent thrombosis in 30% of patients on standard intensity warfarin [22], and included patients with arterial thrombosis in addition to venous thrombosis,. Further research is required to clarify precisely the utility of rivaroxaban in APS treatment.

Similarly studies are ongoing into the potential for Apixaban as a treatment for APS. Much like Rivaroxaban Apixaban is also a specific Factor Xa inhibitor, however, recent results from the ASTRO-APS study have shown issues. The study has been stopped twice, both times due to worse outcomes in the apixaban arm when compared to the control arm, this includes when the dose was increased. The study is now continuing with the exclusion of APS patients with a history of thrombosis [23].

The standard treatment to prevent pregnancy loss in patients with APS is a combination of subcutaneous low molecular weight heparin and oral low-dose aspirin, which gives live birth rates of >70% [24,25]. However, this treatment is not universally effective and these patients may nevertheless suffer increased pregnancy morbidity [24,26]. Hydroxychloroquine (HCQ), an anti-malarial further discussed in section 6.3 below, has been shown to potentially provide further benefit in APS pregnancy [27] and randomised controlled trials are underway [[28], [29], [30]].

Therefore, it is important to develop targeted therapeutics for APS, using our knowledge of how the interaction between pathogenic aPL and β2GPI contributes to the pathogenesis of the disease. This in turn requires a thorough understanding of the function of β2GPI itself in health and disease.

### 1.3 β2GPI *–* more than just APS?

Although β2GPI has a number of proposed roles in both coagulation and complement [31,32], they have been incompletely defined. Research points to β2GPI being able to both up and down regulate serine protease cascades but the mechanisms by which these activities are controlled are currently unknown. A number of studies from various fields have also identified β2GPI in different sites of disease and injury in various different tissues [33]. Zhang et al [34] established that β2GPI is protective in a mouse model of cardiac ischaemia reperfusion injury, building on work by Niessen et al which histologically showed β2GPI was present in human cardiac tissue at the time of ischaemic injury [35]. Furthermore, β2GPI has been found histologically in the placenta of both healthy controls and APS patients [36,37]**,** and pregnant mice [33] demonstrating a role in compromised and healthy pregnancy. In addition, β2GPI has been detected in brain and gut endothelium of mice challenged with lipopolysaccharide (LPS) [33], as well as in the brain of mice undergoing ischaemic brain injury [38], although in this latter study, a faint signal was seen for β2GPI in the brains of sham controls suggesting that β2GPI is present in non-ischaemic brain tissue. β2GPI has even been detected in the retina of patients with age related macular degeneration [39]. At the cellular level, β2GPI is primarily made by hepatocytes, but is reported to be expressed by or bound to different human cells, including decidual endothelium and trophoblasts, cells of the central nervous system [40], monocytes [41,42], neutrophils [43] and cells forming the subendothelial and intima-media regions in human atherosclerotic plaques [44]. In fact, studies in the late 1990s demonstrated that immunisation with β2GPI enhanced atherosclerosis in LDL receptor [44] and ApoE deficient [45] mice, suggesting a central role for β2GPI in pathogenic processes outside thrombosis and pregnancy morbidity. Of note, circulating IgG anti-β2GPI antibodies (aβ2GPI) were detected in both studies following immunisation with a single dose of human β2GPI. Unsurprisingly, given its role in APS, β2GPI has also been detected in blood clots.

Two of the more unexpected roles for β2GPI have been proposed by Dong et al and El-Assad et al suggesting an anti-obesity effect by differentially inhibiting lipogenesis in mice [46] and an anti-bacterial effect specifically in the scenario of gram-negative septicaemia in mice [47]. As research continues it is increasingly clear that β2GPI plays a number of roles in the body. To fully understand the impact of β2GPI in health and disease, a cohesive picture is required.

### 1.4 Review aims

This review aims to bring together the disparate research on the activities of β2GPI in the body and also its various conformations. We focus on the complement and coagulative cascades and how a deeper understanding of the role of β2GPI in current therapies for APS may inform future therapeutic developments.

## 2 Structure and genetics of β2GPI

### 2.1 Open and closed β2GPI

**Fig. 1:**
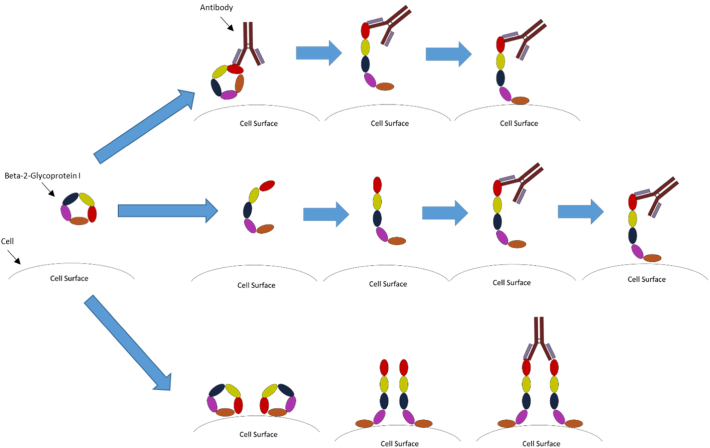
Proposed structural states of β2GPI and the transition to cellular binding. The schematic above demonstrates the potential interactions between cell surfaces, β2GPI and antibodies in APS. The proposed schemes are intensely debated in the field. The top scheme shows antibody binding to a closed β2GPI with this causing β2GPI to open and bind cell membranes. The middle scheme shows β2GPI opening due to environmental factors with an antibody later binding. The bottom scheme shows circular β2GPI binding a cell membrane, opening and dimerization by an antibody. Debate exists as to which of these schemes is the most physiologically relevant.

β2GPI exists in both open (J shaped) and closed (circular) forms [48] (Fig. 1), thus resulting in varying solvent exposures of each of its domains. In particular, it has been hypothesised that both the N-terminal Domain I (DI) and the C-terminal Domain V (DV) are partly hidden in the closed form but become exposed in the open form of the molecule. This is important because DI contains the major epitope region of β2GPI responsible for APS antibody generation [[49], [50], [51], [52]], whereas DV is responsible for binding to cell membranes [[53], [54], [55]]. Despite this research into the structure of the protein, little is known regarding how the structures are maintained, nor which amino acids govern the structure itself.

Agar *et al* [48] found that 90% of the β2GPI circulating in blood is in the closed formation. Thus, if it is true that the open form promotes antibody binding in APS, it is important to understand the conditions which may influence the equilibrium between open and closed forms.

Many proteins exist in the body in a dichotomous state as either active or inactive. Examples include zymogens (such as serine proteases), [56] which require structural cleavage to alter their activity. Another example is tissue factor, which can be encrypted or decrypted by protein disulphide isomerase (PDI) [57]. β2GPI is unusual compared to these examples as there is no obvious enzyme or associating protein to facilitate the structural change between open and closed forms; instead it appears to respond to local environmental stimuli.

Agar et al [48] developed a system by which a change in pH (3.4 or 11.5) and salt concentration (150mM or 1.15M NaCl) can alter the protein structure of β2GPI dramatically. Structural alteration due to shifts in pH suggests that the interactions keeping the protein in its closed form could be heavily influenced by charge. Importantly there are various microenvironments in the body characterised by large changes in pH, oxidative state and oxygen saturation and these could in turn cause β2GPI to assume different structures under normal homeostatic conditions. However, a pH of 11.5 is not seen physiologically. β2GPI is characterised by a high content of lysine residues, mostly located in DV and acetylation of these residues showed a similar conformational change under physiological conditions, supporting the theory/hypothesis that the closed structure is stabilised by electrostatic interactions [58].

A study by Passam et al [59,60] looked in detail at the eleven disulphide bonds found in β2GPI under the assumption that changes in the redox state of these bonds drove structural changes. This led to the discovery of an allosteric disulphide formation in DV. Notably, this disulphide has a typical configuration associated with a middle dihedral strain energy [60], which suggests that it easily undergoes redox changes [61]. Allosteric disulphides can be reduced, which induces larger structural alterations for the protein as a whole. This disulphide conformation is a natural substrate for reduction by the enzyme thioredoxin-1. It has been shown that this reduction is capable of altering the binding properties of β2GPI to antibodies *in vitro* [59]. Further research is required to verify whether enzymatic reduction alters the conformation of the protein *in vivo;,* a comprehensive review of the potential for post-translational redox changes in β2GPI on the potential pathophysiology of APS was published by Weaver et al [62]*.*

Various groups have proposed the existence of intermediate states between fully open and fully closed [63,64]. However, the stability, activity and binding properties of these intermediaries are unknown. It is important to carry out research regarding these potential intermediates to address the role they may play in complement and coagulation and any interaction with pathogenic aβ2GPI. Fig. 1 shows a potential intermediate structure (middle mechanism) and the interaction of antibody/antibody complex with cells.

DV of β2GPI is structurally distinct from the first four domains. As well as a highly distinctive allosteric disulphide bond, DV also contains an unusual loop of lysine residues, which conveys a promiscuous binding character to the whole protein. The lysine loop allows β2GPI to interact with anionic PL and other molecules on cell surfaces, coagulation factors, platelets and complement thus suggesting that β2GPI may have a wide range of functions within the body.

Given the ability of β2GPI to change structure and adopt novel conformations, it is unsurprising that this molecule also has diverse activities in multiple protein cascades. It is currently unknown if this diversity is directly related to the ability of β2GPI to change its structure in these different scenarios.

### 2.2 Glycosylation

Approximately 19% of the molecular weight of β2GPI is composed of glycans [65] and multiple studies have investigated the glycosylation of β2GPI. Clerc et al studied the N-glycosylation of β2GPI and demonstrated that three specific glycans: A2G252, A2G251 and A3G353 were abundant in the protein. Further information on the glycans showed diantennary (two branches) and triantennary (three branches) chains with high levels of sialylation and low fucosylation levels [66]. In the context of APS, patients demonstrated different glycan profiles with a lower amount of triantennary partially sialylated glycans and thus a relative increase in diantennary fully sialylated glycans [66] compared to healthy people.

Glycosylation is a crucial process in the body. Dysregulation of glycosylation is frequently associated with diseases including genetic mutations [67] and autoimmune disorders [68]. Importantly, it has been shown to play a role in the folding of proteins in the body, solubility of proteins [69] and is one of the most frequent post-translational modifications in eukaryotes. The process of glycosylation includes up to 13 different monosaccharides capable of binding eight different types of amino acid and it allows great variation and diversity in proteins. This diversity partially explains the role of glycans as recognition markers and immune modulators whilst also regulating protein turnover and proteolysis.

As an example, factor VIII is an essential cofactor for clotting that is therapeutically available for treatment of haemophilia in a recombinant form. Factor VIII contains 21 glycosylation sites. A study by Kosloski et al. [70] showed that deglycosylation of the protein resulted in significant loss of activity and structural integrity. Similar studies have focussed on the role of glycosylation in factor VII and XII where glycosylation was vital for both stability and activity further confirming that glycosylation may have a significant role in the stability of coagulative enzymes.

It is interesting that glycosylation is important for the stability and activity of coagulative proteins, with which β2GPI can interact, and that differential glycosylation of β2GPI is seen in APS. In combination, these pieces of information raise the possibility that changes in glycosylation pattern alter stability and folding of β2GPI and may play a significant role in the generation of antibodies in APS whilst also potentially playing a role in differential coagulative regulation.

### 2.3 Plasmin cleavage

The interaction between β2GPI and plasminogen/plasmin is complex and bi-directional. Plasmin cleaves β2GPI in a kringle IV domain (a motif shared with plasminogen) in DV at Lys317/Thr318 [71] and research has focussed on the effect of this cleavage on the activity of β2GPI. This study also showed that *in vitro*, the cleaved protein inhibited the proliferation and migration of endothelial cells, an effect not seen with intact β2GPI. As of yet no specific study has detected plasmin clipped β2GPI in the blood of either APS patients or healthy individuals. However, it remains possible that clipped β2GPI does exist *in vivo,* but technical difficulties, low concentration or short half-life make it difficult to detect. Moreover, β2GPI also binds to tissue plasminogen and is a cofactor for its activation to form plasmin [72]. Plasmin-cleaved β2GPI binds plasminogen less well than intact β2GPI suggesting a negative feedback loop. Lopez-Lira et al [73] hypothesised that significant homology between lipoprotein Lp(a) (a known ligand of β2GPI) and plasminogen may be the reason for β2GPI targeting plasminogen. This group also showed a dose-dependent increase in the production of plasmin as levels of β2GPI were increased.

A study in 2001 by Guerin et al [74] demonstrated the ability of heparin to increase the plasmin-mediated inactivation of β2GPI through upregulating cleavage in the kringle domain. The study assessed plasmin-cleaved β2GPI for its ability to bind both heparin and cardiolipin. For both binding partners, affinity was found to be significantly diminished post cleavage.

Matsuura et al studied the effects of plasmin cleavage on the antigenicity of β2GPI [75], confirming the loss of cardiolipin binding in the presence of autoantibodies but also suggesting through molecular modelling that novel hydrophobic and electrostatic interactions in DV are generated in the process of cleavage. This proposal requires significant structural work for confirmation.

The effect of β2GPI on the activation of plasmin and the ability of plasmin to prevent β2GPI binding to the cellular surface suggest a complex and intricate feedback mechanism that may have antithrombotic and fibrinolytic implications. This is an interesting mechanism by which β2GPI can act as a regulator of coagulation both in health and disease, particularly in APS where binding of autoantibodies could disrupt the mechanism.

### 2.4 Genetic variations

A number of different genetic variants of β2GPI have been described in human serum. They were first identified by Richter et al [76] who conducted isoelectric focussing and immunoblotting of sera from 400 healthy donors. The results revealed six genetic phenotypes whilst 44 family studies demonstrated the genetic linkage. Treatment of these samples with neuraminidase and endoglycosidase F (to remove glycans) failed to resolve the profiles with all six spots still being identifiable, suggesting the genetic variation is not glycan related. Of the six phenotypes described, four variants were confirmed by Cleve et al in an African cohort in 1992 [77]. The theory for a genetically driven molecular structural variation was first proposed by Sanghera in 1997 [78] who determined missense mutations causing two of the variants, with the Asn88 allele being especially high in black subjects.

Kamboh et al [79] studied two common mutations in DV at positions 306 and 316 and found that patients homozygous for these mutations or with compound heterozygote presentation had reduced binding of β2GPI to phospholipids. The patient numbers in this study are not reported, thus it is hard to draw strong conclusions from this, although a follow-up study by the same group was more convincing and included the prevalence of antibodies capable of recognising a complex of β2GPI and cardiolipin (CL). In a study of an African population (n=755), the background positivity for the anti-CL/aβ2GPI complex antibody was 50% (compared to 10% for a US baseline level) whilst the prevalent genetic mutations contained polymorphisms in the third domain of β2GPI [80]. Reconstruction of the alleles found in this study in order to express recombinant β2GPI showed altered binding to anionic phospholipids.

Mehi et al suggested that the levels of β2GPI in plasma were influenced by genetic control through one of three alleles (APO1-3); the APO2 allele [81]. Amongst the factors that were suggested to influence plasma levels of β2GPI were age, cholesterol levels, triglyceride levels and HDL-cholesterol levels but only in women. Further study of the genetic variation demonstrated lower levels of plasma β2GPI associated with the APO3 allele. The greatest variation of plasma β2GPI level was seen in people with the APO3*W missense mutation at codon 316. This study carried out in Pittsburgh USA, studied only white patients and did not assess Hispanics or any other ethnicities; thus its ability to be extrapolated to other races and the wider population as a whole is limited. A study in 1998 [79] attempted to associate genetic alleles of β2GPI with structural variation. Although genetic research has not elucidated novel structures of β2GPI, it has shown that minute genetic changes may alter the ability to generate antibodies to β2GPI.

## 3 Functional roles of β2GPI

The two main functions of β2GPI to regulate complement and coagulation are relevant to the pathogenesis of APS. The ability of β2GPI to regulate coagulation is complex since it has antithrombotic (both anticoagulant and antiplatelet) as well as procoagulant effects.

### 3.1 Beta-2-glycoprotein: anticoagulant, antiplatelet and procoagulant effects

The balance between the opposing effects of β2GPI in coagulation is dependent on the surrounding environment. The varying roles are summarised in Table 1. The effects of β2GPI in coagulation can be subdivided into direct and indirect. Indirectly, β2GPI can exert an anticoagulant effect through downregulation of thrombin generation whilst its indirect coagulant effect is shown through mechanisms including inhibiting activation of protein C and disrupting the anticoagulant Annexin V shield. Direct methods of influencing coagulation include inhibiting the thrombomodulin complex (Procoagulant) and binding thrombin to downregulate its activity (anticoagulant). β2GPI also regulates platelet activation [82]. The fine balance between these interactions is not completely understood and requires significant research to understand what regulates the pro- and anticoagulative effects of β2GPI in health and disease.

**Table 1:** Summary of the role of β2GPI as both a pro- and anticoagulant factor.

Anticoagulant, antiplatelet and profibrinolytic	*Evidence derived in vivo/in vitro?*	Procoagulant	*Evidence derived in vivo/in vitro?*
β2GPI can bind the ApoER2’ receptor. ApoER2‘ binds Factor XI on platelets [83] leading to thrombosis. In turn, β2GPI competitively inhibits this mechanism.	*In vitro*	Various studies have proven that many of the Lupus Anticoagulant (LA) effects seen in APS patients are β2GPI dependent [[84], [85], [86], [87]]. This term lupus anticoagulant is misleading as this effect actually causes increased coagulation *in vivo* whilst *ex vivo* the effect is anticoagulant.	*In vitro*
ApoER2’ on platelets is required for the immobilisation and activation of protein C [88] and thus regulation of thrombin generation. β2GPI also competitively inhibits this process.	*In vitro*	β2GPI interacts with Annexin V, inhibiting the anticoagulant effect of Annexin V [89].	*In vitro*
β2GPI can directly inhibit thrombin activation [90].	*In vitro*	β2GPI demonstrated procoagulant activity by inhibiting activated protein C [91] leading to impaired thrombin generation [92].	*In vitro/in vivo*
β2GPI prevents plasminogen activator inhibitor 1 from acting upon tissue plasminogen activator, thus downregulating its fibrinolytic activity [90].	*In vitro*	Activated protein C (aPC) is created by an interaction of thrombomodulin and thrombin at a rate of 1000 fold in comparison to thrombin production alone, β2GPI has been shown to inhibit this interaction, reducing the production of the anticoagulant aPC [93].	*In vitro*
In the presence of β2GPI, platelet aggregation through ADP is severely impaired [94].	*In vitro*	β2GPI could inhibit the inhibition of thrombin by a combination of heparin and its cofactor [95].	*In vitro*
β2GPI binds Factor XI and in turn prevents activation by thrombin thus preventing the formation of a positive feedback loop [32,96].	*In vitro*		
β2GPI interacts with platelets preventing generation of FXa [97].	*In vitro*		

In patients with APS, aβ2GPI form complexes with β2GPI [98,99]; however, how this influences the functional effects of β2GPI in patients is not fully understood. Patients with APS develop clots suggesting that aPL binding to β2GPI negatively alters the anticoagulant processes or increases the procoagulant effects. Studies have identified targeting platelets and disrupting annexin shields as mechanisms of pathogenesis in APS, but considerably more research is needed to probe the influence of aPL on β2GPI regulation of coagulation. The potential generation of circulating aβ2GPI/β2GPI is debated in the APS field, however, recent research has shown that circulating immune complexes of IgA subclass have been detected in the serum of APS patients and are associated with thrombotic events [98,99]. Circulating IgG and IgM complexes with β2GPI have also been recently associated with non-criteria clinical manifestations of APS [100]; this suggests that, although APS is not classically characterised as a disease of circulating immune complexes there may be an emerging role for them in its pathogenesis.

In addition to the coagulation cascade, β2GPI also influences and regulates other systems within the body, most notably complement. This ability to be regulatory in both the complement and coagulative pathways is supported by recent research demonstrating potential ‘cross talk’ between the two systems [101].

### 3.2 Beta-2-glycoprotein and complement

Given that the structure of β2GPI includes Complement Control Protein (CCP)-like domains it is unsurprising that it also plays a role in the complement regulation [102]. However, the extent to which β2GPI physiologically regulates complement is unknown. Gropp et al propose that β2GPI has effects as a cofactor for complement inhibition [103], suggesting that this inhibitory effect is brought about *via* β2GPI in its open form in the presence of C3. They suggest the binding of β2GPI to C3 facilitates the subsequent binding of factor H thus enhancing degradation of C3 to C3i by factor I. It has also been suggested that C3 cleavage by factor I in the absence of factor H is possible in the presence of β2GPI [103]. This ability to bypass factor H binding is unique to β2GPI and represents a significant role in regulation of complement.

Similarly, β2GPI has also been referred to as a component of the innate immune system due to its ability to bind to and neutralise lipopolysaccharide (LPS). This effect was described by Agar et al. [104] who carried out a series of experiments looking at the potential role of β2GPI in the response to LPS in Gram-negative septicaemia. Using surface plasmon resonance and electron microscopy, they demonstrated that LPS can bind β2GPI *via* DV and that this leads to opening of the β2GPI structure. Either whole β2GPI or DV alone could inhibit LPS-induced release of tissue factor (TF) from monocytes or endothelial cells in culture. When 23 healthy volunteers were infused with LPS they developed fever and tachycardia and there was a mean 25% fall in the serum level of β2GPI that lasted at least 24 hours. The authors suggested that this fall was due to β2GPI engaging and removing LPS and this hypothesis was supported by the finding that volunteers who had lower β2GPI before administration of LPS developed higher fevers and more release of inflammatory cytokines such as tumour necrosis factor, interleukin 6 and interleukin 8. Lastly, in a study of patients on the intensive care unit, 35 patients who developed Gram-negative septicaemia had lower β2GPI levels than 36 who did not – but these levels returned to normal after recovery from sepsis. These results are interesting since LPS can stimulate both the complement and coagulation cascades so this role of β2GPI is another potential way in which those systems can be co-regulated [104].

The cleavage of C3 and C5 by FXa and thrombin is increasingly acknowledged as a potential mechanism through which alternative activation of complement occurs. β2GPI also has the ability to alter both thrombin and FXa activity either through preventing inhibition of FXa/thrombin by its natural regulator or alternatively through preventing activation of both enzymes from their respective zymogens. Thus, the ability to prevent activation of FXa or thrombin may be another route by which β2GPI can dampen complement activation in patients.

The ability of β2GPI to alter plasmin generation has been discussed in section 2.3. In the context of complement it is worth noting that plasmin is capable of generating complement through cleavage of C3 and/or C5. This implies that this self-regulating interaction between plasmin and β2GPI may have a role in complement activation too.

Complement has been shown to play a crucial role in the pathogenesis of APS and several comprehensive reviews focus on this [105,106]. In a series of experiments in a murine model of APS pregnancy, Salmon and co-workers showed that infusing large amounts of IgG from patients with APS to mice early in pregnancy caused a significant decrease in the number of viable foetuses [107]. This effect was reduced in complement-deficient mice [108] or in the presence of complement inhibitors [109]. They proposed that this complement-dependent mechanism for pregnancy loss in APS could be relevant to the efficacy of heparin in preventing APS-induced pregnancy loss. Comparison of the effects of two anticoagulants, heparin and hirudin, in this model showed that only heparin blocked the pathogenic effect of the IgG from patients with APS and only heparin blocked the activation of complement [107]. Thus, this group suggested that complement activation in the placenta plays a major role in APS pregnancy morbidity. The role of complement in adverse pregnancy outcomes was also studied by Kim et al. who showed increased levels of complement breakdown products in the serum of pregnant patients with SLE and/or APS [110]. Other work has shown that endometrial biopsies from patients with APS had reduced expression of complement-regulatory proteins [111]. Other groups have also demonstrated the involvement of complement in both thrombosis and pregnancy loss models of APS [112,113]. However, complement modulators are not commonly used in the treatment of APS, though there have been reports of therapeutic use of the monoclonal anti-C5 antibody eculizumab with occasional successes in cases of catastrophic antiphospholipid syndrome (CAPS) [[114], [115], [116], [117]] effectively preventing re-thrombosis in some patients [[118], [119], [120]]. Although promising, the small number of cases of CAPS limits the possible extrapolation of these studies to a more widespread guideline and as such these successes have yet to be reflected in the best practice guidelines for CAPS [121].

APS patients frequently present antibodies (anticardiolipin, aβ2GPI) that can fix complement and also dysregulate coagulation, these two cascades have been shown to cross talk in health and disease [[122], [123], [124]]. Further research into the subclasses of antibodies in APS have shown they should be capable of fixing complement [[125], [126], [127], [128]] suggesting complement activation may play a major role in APS. This ability to regulate both complement and coagulation directly is found in only three proteins: β2GPI, thrombomodulin and C-reactive protein (CRP).

## 4 Beta-2-glycoprotein, thrombomodulin and C-reactive protein

Thrombomodulin and β2GPI both interact at very similar points of the coagulation and complement pathways whilst CRP plays a different role. Production of inhibitory factors for the complement cascade is driven by CRP whilst it can also act as a prothrombotic protein in the presence of platelets [129], specifically through blood coagulation factors and by altering the fibrinolytic system [130,131]. Interestingly, both β2GPI and thrombomodulin exert their effects as cofactors for other processes. As shown in Table 1, thrombomodulin can upregulate thrombin mediated activated PC (aPC) production approximately 1000-fold, leading to an anticoagulant effect. β2GPI can interfere with the formation of this thrombin/thrombomodulin complex downregulating the effect of thrombomodulin and thus aPC [91]. Independently of this, the structure of β2GPI has been shown to be important in altering coagulative processes including thrombin generation [132]. Conversely, thrombomodulin can upregulate the cleavage of C3b to C3i which is mirrored by the activity of β2GPI, however, β2GPI achieves it more efficiently as it removes the necessity for factor I as a cofactor for factor H. The complement inhibitory activity of both β2GPI and thrombomodulin both link with CRP which upregulates the production of inhibitory factors I and H.

**Fig. 2:**
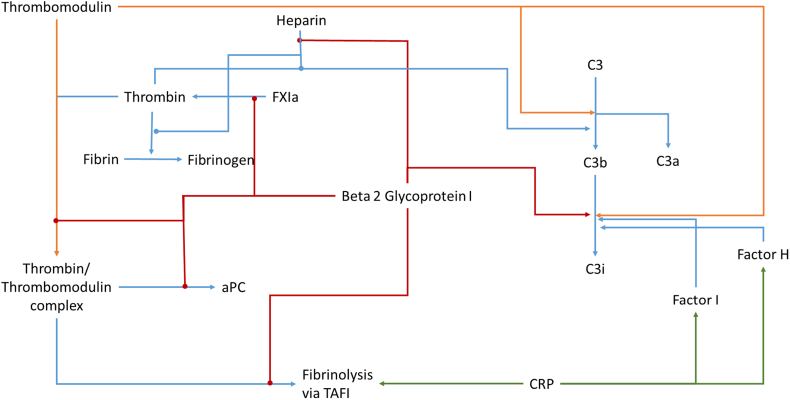
Coagulation and Complement Interactions. This diagram shows the varying contrasting interactions of CRP, thrombomodulin and beta-2-glycoprotein I. Red arrows indicate upregulation in the presence of β2GPI whilst red arrows with a circular ending indicate inhibition in the presence of β2GPI. Orange arrows represent upregulations in the presence of thrombomodulin and green arrows upregulation in the presence of CRP. The blue lines represent physiological interactions which may be altered by the proteins. (For interpretation of the references to color in this figure legend, the reader is referred to the web version of this article.)

The complex interplay between β2GPI, CRP and thrombomodulin is shown in Fig. 2. Crucially, β2GPI is the only molecule which can either upregulate or downregulate either pathway, directly or indirectly. Thrombomodulin and CRP are less versatile as they only upregulate either pathway. Thus, agents targeting functions of β2GPI and its interaction with aβ2GPI could present a promising avenue for the treatment of APS or other coagulant or complement based disorders.

## 5 β2GPI as an autoantigen – development of new diagnostic tests

### 5.1 Why do aβ2GPI antibodies develop?

The production of autoantibodies requires a loss of peripheral immune tolerance. The high serum concentration (0.2 mg/ml) [133] of β2GPI would suggest that it should be unusual for immunological tolerance to β2GPI to be broken. There are several theories to explain how tolerance is broken in APS. There have been suggestions that it may be due to a higher amount of β2GPI in patients or as a result of a multi-factorial process. This suggests that patients have an intrinsic risk of developing autoimmune antibodies (for example, as a result of genetic susceptibility) before a second process such as higher levels of β2GPI, or β2GPI in an alternative structure, exposes excessive cryptic antigen thus allowing for a loss of tolerance and subsequent autoantibody generation. The potential for increased amounts of β2GPI in patients who develop antibodies has been studied; however a lower threshold for production of antibody has yet to be proven [134]. An alternative suggestion is that antibody development may be *via* an impaired clearance of apoptotic material decorated with β2GPI [135], as described in systemic lupus erythematosus (SLE) for other antigens [[136], [137], [138], [139], [140]]. Alternative views claim that the amount of β2GPI which is in the open state, thus exposing the antigen, is different in APS patients when compared to individuals who do not have the disease [141]. One group has gone so far as to show β2GPI is presented in an unusual fashion on MHC molecules [37], whereby the whole protein rather than cleaved peptides is bound, whilst others show that the passage of β2GPI through immune cells is far from straightforward including differences in intracellular trafficking [142]. Rather than β2GPI being proteolytically digested and presented as normal, β2GPI stagnates in the late endosome and is transported to the cellular surface for presentation to autoreactive CD4+ T cells in an MHC II restricted manner potentially resulting in presentation and antibody generation [37].

One of the most prominent targets for pathogenic aPL is the endothelium, which forms the interface between blood and the surrounding tissue [143]. Importantly, under inflammatory conditions, and particularly in the presence of IFN-γ, endothelial cells (EC) can upregulate MHC II and act as non-classical antigen presenting cells (3), a process likely to play a critical role in autoimmunity [144]. Indeed, β2GPI co-localised with MHC II has been detected in the endothelium of decidual biopsies from APS patients, while non-APS biopsies stain for β2GPI but not MHC II, suggesting that class II expression and hence antigen presentation is induced in APS specifically (34). Importantly, the interaction of β2GPI with the endothelium is not dependent on MHC II, as the protein can bind EC *via* a number of different molecules including anionic structures such as heparan sulfate, annexin A2 [145], Toll like receptor 4 [146,147] and ApoER2 [148] (schematic for all binding partners in the following reference [143]). As mentioned previously, β2GPI is abundantly found in sub-endothelial regions of atherosclerotic plaques, possibly due to its ability to interact with the aforementioned molecules, and localises in close proximity to CD4+ T cells [43]. Benagiano et al eloquently demonstrated that plaques from both primary [149] and SLE-associated [150] APS patients contain a large proportion of β2GPI-reactive CD4+ T cells capable of producing inflammatory cytokines and activating autologous B cells to release immunoglobulin. A major cytokine released by these β2GPI-reactive T cells is IFN-γ ([149,150] that can drive endothelial MHC II upregulation[144]. All this evidence points towards an attractive model for antigen ‘presentation’ by EC, facilitating aPL binding to EC but also promoting autoantigen presentation and autoreactive T cell activation, resulting in the breakdown of tolerance.

In the study by Benagiano et al, plaque derived β2GPI-reactive T cells were found to be considerably more abundant than peripheral β2GPI-reactive T cells [149]. Of interest, >80% of β2GPI-reactive plaque derived T cell clones recognised DI [149], while in the periphery, separate reports suggest that most β2GPI-reactive CD4+ T cells recognise DIV-V [151]. These disparate results may simply reflect a change in T cell epitope specificity after homing into their target tissue, possibly due to a conformational change in tissue-bound β2GPI exposing the DI epitope.

### 5.2 β2GPI and diagnostic tests in APS

Several groups have studied the prognostic and diagnostic value of aβ2GPI in patients with APS [[152], [153], [154], [155], [156]]. It has been demonstrated that these antibodies correlate strongly with thrombotic events [49,[157], [158], [159], [160], [161], [162]], as do LA assay results [84,[163], [164], [165], [166], [167], [168], [169]]. However, it is unusual for patients to show sole positivity for aβ2GPI. Equally, there is a group of patients who have clinical manifestations of APS but test negative in all of the current criteria assays – sometimes called seronegative APS (SN-APS) [170,171]. There is therefore interest in developing new assays to aid both diagnosis and risk stratification in patients with APS, and the primacy of β2GPI as the key autoantigen has informed development of these tests – particularly IgA aβ2GPI [172] and anti-DI antibodies [[173], [174], [175]]. The inclusion of anti-DI antibodies has particular significance when considering the differences in diagnostic value from antibodies targeting DV which are believed to be non-pathogenic [176,177]. Andreoli et al carried out a study in which serum from 159 subjects with persistently positive medium or high-titre IgG anti-β2GPI was tested by ELISA for both IgG anti-DI and anti-DIV/V antibodies. The subjects were fully characterised clinically into the following groups: 56 with thrombotic PAPS (primary APS), 39 with pregnancy morbidity, 31 with purely obstetric PAPS, 42 with autoimmune rheumatic disease but not APS and 30 aPL carriers with no autoimmune rheumatic disease or APS. This last group of healthy aPL-carriers had higher anti-DIV/V but lower anti-DI than the other groups. Thus a ratio of anti-DI to anti-DIV/V of > 1.5 was associated with autoimmune rheumatic disease, but not specifically with APS or thrombosis [178].

Pierangeli *et al* showed the pathogenic potential of IgA in a murine model [179] and proposed the potential for IgA aβ2GPI positive patients to develop APS even in the absence of IgG and IgM aβ2GPI. Furthermore, Shen *et al* showed clinical significance for IgA aPL in a study of 472 patients [180] in predicting thrombotic events. This finding was also highlighted in a review by Andreoli et al who outlined both raised levels of IgA aβ2GPI in SLE patients who develop APS and a significant association with thrombosis [181]. Pericleous et al found that IgA aβ2GPI associates with thrombosis and also highlighted the added diagnostic value of testing IgA anti-DI [182]. Furthermore, Murthy et al [183] found aβ2GPI IgA titres correlated with clinical features of APS and highlighted the role of IgA aPL directed to the 4^th^ and 5^th^ domain of β2GPI in patients with APS.

A recent study of 40 SN-APS patients found positivity for either IgA aβ2GPI or anti-DI in 10% of patients with SN-APS [184]. There is a growing call for these non-criteria antibody tests to be included in APS diagnosis, and future research should investigate the added value of such tests in management of patients with this syndrome.

The issue of validity of different diagnostic tests in APS is controversial. Currently there is significant difficulty in standardization of the testing for aβ2GPI, sources of β2GPI are non-identical and no international reference material exists for aβ2GPI. This is a problem which has been discussed at length [[185], [186], [187]] but at the time of writing has not been resolved despite taskforces and concerted efforts from groups worldwide [188,189].

### 5.3 Anti-domain I antibodies

As discussed in section 2.1, it is believed that DI and DV are associated in the closed form of β2GPI, hiding the dominant epitope for aPL antibodies in the R39-G43 region of DI. It is important to be aware that antibody binding can also be altered by substitutions elsewhere in the sequence of DI [[190], [191], [192]]. Given the presence of a dominant epitope in DI, several groups have published results looking at anti-DI positivity in patients with APS.

Numerous groups have examined the potential for anti-DI antibodies diagnostically. It has been shown in some studies that that adding IgG or IgA anti-DI to the diagnostic criteria increases the sensitivity of the criteria [182,193,194]. Other reports, however, concluded that the IgG anti-DI assay did not add to the value of current criteria assays in predicting thrombosis [195,196]. In a meta-analysis of 11 studies including 1218 patients with APS, 318 patients with SLE, 49 asymptomatic aPL-positive individuals and 1859 healthy controls, Radin *et al* reported that 45.4% of patients with APS were positive for anti-DI. Studies that looked at association between anti-DI-positivity and risk of thrombosis found such an association, with odds ratios ranging between 2.5 and 4 [197].Others have argued the range of discrepancies between studies due to methodological differences means there is little clinical value to including anti-DI in testing until standardized calibrators are available [198]. Recent research has begun to show that aβ2GPI results do not directly reflect associated aDI results from the same patients, with different specificities of subclass being detected [125]. Some groups are advocating far more wide-ranging studies into the utility of aDI diagnostically [193], however, a lack of cohesive methodology and specificity is holding back these efforts.

### 5.4 Other non-criteria APL

Although this review concentrates on the role of β2GPI and anti-β2GPI antibodies in APS, it is important to recognize that other non-criteria aPL have been investigated as possible additions to the diagnostic armamentarium for APS. Antibodies to phosphatidylserine (anti-PS), prothrombin (anti-PT) and the phosphatidylserine/prothrombin complex (anti-PS/PT) have attracted particular interest. The studies regarding these antibody tests were reviewed thoroughly by a taskforce of the 14^th^ International Congress on Antiphospholipid Antibodies [172]. Overall, the evidence did not support any value for testing anti-PT but supported further studies of anti-PS and anti-PS/PT.

In a systematic review of 20 studies including 5992 patients, Radin et al reported that a median of 55% of patients with confirmed APS were IgG anti-PS-positive (35% IgM anti-PS-positive) and that these prevalence figures were significantly higher than those seen in patients with SLE and no APS (IgG anti-PS in 22%, IgM anti-PS in 14%). From these data, however, it was not possible to establish an independent association between anti-PS-positivity and either vascular thrombosis or pregnancy morbidity.

To eliminate variation in results due to different anti-PS/PT assays being used by different research groups, a collaborative multi-centre study looked at samples derived from different patient cohorts but all tested at a central facility using two different IgG anti-PS/PT ELISA [199]. Results from the two ELISA showed strong correlation. In an initial study of 247 subjects from 8 centres (126 APS, 73 autoimmune disease controls, 48 healthy controls) the prevalence of IgG anti-PS/PT positivity in patients with APS was 58%. In 204 patients who gave concordant results in both ELISA, positivity for IgG anti-PS/PT gave a sensitivity of 51% and specificity of 91% for APS. Subjects positive for IgG anti-PS/PT were significantly more likely to have had vascular thrombosis (odds ratio 11.0, 95% confidence interval 3.8-31.3) or obstetric APS (odds ratio of 10.6, 95% confidence interval 3.5 to 32.1). A replication study in 214 subjects (96 APS, 67 autoimmune disease controls, 51 healthy controls) from five new centres gave very similar results for thrombosis – sensitivity and specificity for APS 47% and 88% respectively, odds ratio for vascular thrombosis 11.3 (95% CI 4.2 to 30.0) but positivity for IgG anti-PS/PT was not associated with obstetric APS in the replication cohort [199].

Evidence that adding the IgG anti-PS/PT test to the standard criteria assays may be of value comes from a Japanese study by Otomo et al [200] in which samples were subjected to five different LA assays and six different ELISAs – IgG and IgM for each of aCL, anti-β2GPI and anti-PS/PT. The results of all the tests were combined into a numerical score designated aPL-S. The predictive value of aPL-S was assessed in 411 subjects who were followed prospectively after their aPL-S was measured. Of these, 32 developed thrombosis and these patients had significantly higher aPL-S scores at the beginning of follow-up (P=0.012). Patients with aPL-S > 30 had fivefold higher risk of developing thrombosis than those with lower aPL-S scores.

## 6 Beta-2-glycoprotein I as a therapeutic target

### 6.1 Indirect targeting of functional effects of aβ2GPI

Various attempts at targeting aβ2GPI either directly or indirectly as a treatment for APS are currently under development.

In a recent study, four patients with APS unresponsive to conventional anticoagulation therapy, were treated with eculizumab (a C5 inhibitor). Thrombosis was not an outcome of the study, which instead reported increases in platelet count (initially reduced in all four patients ranging between 18000 and 85000 per ml). It is possible, however, that the C5 inhibition may have been acting on co-existent idiopathic thrombocytopenia rather than on aβ2GPI induced thrombocytopenia [201]. Nevertheless, it has been suggested by Gropp et al [202] that eculizumab may act by blocking the pro-complement activity of the aβ2GPI/β2GPI complex and thus compensating for the dysregulation of β2GPI-complement interactions in APS.

Heparin and its variants have also been shown to target β2GPI with Kolyada et al [203] characterising the binding site of fondaparinux on β2GPI as an amino acid sequence in DV. This study further evaluated the effects of fondaparinux on the binding of aβ2GPI/β2GPI complexes to cardiolipin showing that binding was still possible whilst competitive binding with heparin was inconclusive. A study by Guerin et al showed that heparin binding to β2GPI prevents binding to cellular surfaces [74], although interestingly, the same was not true of fondaparinux. Neither study examined the effect of heparin or fondaparinux on the ability of β2GPI to form antigen/antibody complexes. Although these therapies target aβ2GPI, they do so indirectly.

### 6.2 Direct targeting of β2GPI or aβ2GPI

One new potential therapeutic is named A1-A1, a peptide of approximately 40 amino acids [204] that utilises a synthetic dimer of ligand binding domains from ApoER2 [205] to target the fifth domain of β2GPI and prevent binding to cell surfaces. The two A1 molecules are bound by a flexible linker allowing binding to β2GPI in the fluid phase. The stability of the A1-A1 linkage has been shown across 15 days in an accelerated stability study with good success [205] whilst inhibition of binding to cardiolipin has been shown with an improved mutant of A1 [206]. Further experiments included inhibiting the thrombotic potential of aβ2GPI in murine models [207] and a reduction of blood pressure in mice [208]. The group developing this potential therapeutic has since proven dimerization of DV of β2GPI is sufficient to generate an increase in stimulation of a monocyte cell line, presumably to show this is also inhibited by the administration of A1-A1.

This therapy shows significant promise, however, due to the small size of the peptide dimer (~8kDa) it is likely it will need biochemical modification prior to future use in humans, this suspicion is reflected in the mouse model used showing reduced thrombus generation 10 minutes after infusion of A1-A1 [207].

Another potential therapeutic is a cytomegalovirus capsid peptide known as TIFI which is approximately 20 amino acids long and which shows strong homology to a 15-mer from DV of β2GPI. TIFI was shown to inhibit the thrombogenic properties of IgG antibodies purified from APS patients [209] in a mouse model. A further study by the same group confirmed this action [210] was through the targeting of the 5^th^ domain of β2GPI. TIFI was successfully tested for its ability to inhibit murine foetal loss [211] and it was further shown to be protective on endometrial endothelial cells [13]. These studies show significant potential for TIFI as a therapeutic for APS.

In addition, recombinant Domain I of β2GPI expressed in bacteria has been a proposed as a novel therapeutic agent [191] with the aim of generating a soluble form to bind aβ2GPI antibodies and thus prevent formation of the aβ2GPI/β2GPI complex. Initial research was promising with both wild-type DI and a mutant form containing two point mutations shown to inhibit binding of antibodies from APS patients to β2GPI in an ELISA. Both forms were able to block ability of these antibodies to promote vascular thrombosis in a mouse model [212]. In addition, recombinant DI was also found to reduce caspase 3 production in an aPL-based model of cardiac injury [213]. In order to circumvent the problem of small size of DI, this research group has recently described the production of PEGylated DI and have shown this molecule retains the ability to inhibit IgG antibodies purified from blood of patients with APS in both binding and thrombogenic assays [214]. Anti-thrombotic activity *in vivo* was seen in an acute mouse model at several doses of DI [214] suggesting great therapeutic potential. Further research has been carried out by other groups including a recent study suggesting Domain I may be effective *in vivo* in a chronic model of APS [215].

The group of Agostinis et al. have worked on developing a potential therapeutic for APS: a non-complement fixing antibody to β2GPI[216]. This molecule has significant potential. It is a single chain fragment variant (scFv) that has shown the ability to decrease the pathological effects of aβ2GPI *in vivo* in mouse models through displacing patient-derived antibodies [216]. Although this is undoubtedly an interesting and novel approach to an APS therapeutic, it remains the only current publication for this agent and as such it is hard to gauge the full clinical utility of the scFv in APS, however, it has significant potential to be explored in this remit.

Although there are several technologies aimed at specifically targeting β2GPI in production, these are very far from clinical practice as yet and still require safety and efficacy studies in humans before we can know the potential of these agents.

### 6.3 Hydroxychloroquine in APS

The role of the antimalarial, hydroxychloroquine (HCQ), in the management of APS has long been extensively debated in the literature [217] and the drug is commonly used in the management of SLE (which is frequently associated with secondary APS). Previously, Nuri *et al* demonstrated that it plays a role in lowering aPL and preventing recurrent thrombosis in patients with lupus. In addition to observing a reduction in IgG anti-cardiolipin, a significant decrease in both IgG and IgM aβ2GPI was observed following treatment with HCQ thus suggesting that this treatment may have an immunomodulatory effect [218]. The exact mechanism through which HCQ conveys this benefit is still poorly understood, however, it has been suggested that complement plays a key causative role in placental ischaemia and abnormal foetal brain development in APS. Using radioacitve iridium (^111^In) labelled aPL antibodies, Bertolaccini *et al* investigated this interaction in a murine model of obstetric APS and found that, although HCQ did not affect aPL binding to the foetal brain, it did prevent activation of complement. Notably C5a levels from both APS patients and the murine model were lower after treatment with HCQ, suggesting that it may demonstrate benefit through inhibiting complement activation [219]. Further studies in murine models have recently been conducted by Miranda *et al*, who focused on the way in which aPL antibodies promote endothelial dysfunction in thrombotic APS. The study centred on comparing the difference seen in mice that had been inoculated with human aPL antibodies that were in turn treated with and without HCQ. In those treated with HCQ, a reduction in thrombosis formation, reduced thrombin generation time and improved endothelial-dependent relaxation was observed. HCQ was also found to modulate endothelial nitric oxide synthase [220]. HCQ has also been shown to improve endothelium-dependent dilatation after three weeks of treatment in an APS mouse model [221]. In a study of 22 patients with APS treated with HCQ (200 mg/day for three months) it was shown that it resulted in a reduction in soluble tissue factor levels, which may in turn convey benefit in reducing vascular events [222].

Clinically, the benefits of HCQ in high risk APS pregnancies was demonstrated by Ruffatti *et al*, who found that from a total of 196 pregnant mothers with APS, significantly higher live birth rates were seen in those taking hydroxychloroquine. Furthermore, HCQ conferred greatest benefit to mothers without a history of previous thrombosis [223]. The role of HCQ in reducing thrombotic complications of APS has been widely evaluated [224]. A study by Erkan *et al* aimed to evaluate the role of HCQ in primary prevention of thrombosis in aPL-positive patients in the absence of other systemic autoimmune disease. Unfortunately the study was terminated early due to low recruitment rates and the authors concluded that the efficacy of HCQ in these cases could not be fully assessed, thus highlighting the challenges of achieving reduced thrombosis as a primary outcome in clinical trials in APS [225].

## 7 Summary and future directions

β2GPI has been recognised as the key antigen targeted by pathogenic antibodies in patients with APS for many years. It is only more recently that the unique nature of this glycoprotein, both in structure and function, has been explored in detail. β2GPI can take two main structural forms, open and closed, which may differ in exposure of the key antigenic epitope on DI. The conformational dynamic of this protein (i.e. the shift between open and closed forms) is controlled by post-translational modification and changes in pH. Intermediate forms between open and closed may also exist. Functionally, β2GPI is unique in being able to regulate both complement activation and haemostasis in either direction. These actions of β2GPI can be influenced by aβ2GPI antibodies present in patients with APS and may be potential therapeutic targets. Assays to measure levels of antibodies to β2GPI and to DI show promise in improving diagnosis and risk stratification of patients with APS. A number of proposed therapeutic agents that target β2GPI/aβ2GPI interactions are in development.

## 8 Conclusions

β2GPI is a unique protein capable of regulating both complement and coagulation cascades and maintaining or altering haemostasis. It is present at the site of various disease processes and exists physiologically in at least two structural forms. Little is known relating structure of β2GPI to its function and the nature of intermediate structures between open and closed β2GPI is poorly understood. β2GPI stands at the junction between the complement and coagulation cascades and could play an important role in cross-talk exhibited by these two key physiological systems. The presence of aβ2GPI antibodies in APS could modify these interactions contributing to the pathogenesis of thrombosis and pregnancy morbidity. It is imperative that further research is conducted into better understanding this unique protein that is capable of up and down regulating both the complement and coagulation systems as well as being a key autoantigen in an important autoimmune disease.

## 9 Practice points

• The co-regulation of the complement and coagulation cascades both in disease and haemostasis is an important process to which β2GPI may be contributory.
• In APS the most important pathogenic antibodies target β2GPI rather than binding phospholipids directly.
• The presence of IgM and IgG aβ2GPI measured by ELISA is one of the classification criteria for diagnosis of APS.
• Newer assays such as IgA aβ2GPI and measurement of antibodies to DI may be used in diagnosis and management of APS in future.
• Current therapies for APS, notably heparin, may work partially through an effect on interaction of β2GPI with the complement cascade.
• New therapies that target either DI or DV of β2GPI are being developed, but chemical modification such as PEGylation will be needed to improve pharmacological properties.

## 10 Research agenda

• Do intermediate structural forms of β2GPI exist *in vivo*, how stable are they, and what are their properties?
• Can β2GPI be targeted in clotting and complement disorders to alter regulation therapeutically?
• Can anti-complement agents such as eculizumab be used in treatment of APS?
• Are the novel small molecules being developed to target DI and DV viable therapeutic agents for APS?
• Are the benefits of measuring anti-DI and IgA aβ2GPI levels sufficient to add these assays to the classification criteria for APS?

## Declaration of Competing Interest

TM, CP, IG and AR are inventors on the patent for Domain I.
